# A prospective cohort study about the effect of repeated living high and working higher on cerebral autoregulation in unacclimatized lowlanders

**DOI:** 10.1038/s41598-022-06270-z

**Published:** 2022-02-15

**Authors:** Laura C. Graf, Sara E. Hartmann, Mona Lichtblau, Lara Muralt, Patrick R. Bader, Ivan Lopez, Jean M. Rawling, Silvia Ulrich, Konrad E. Bloch, Marc J. Poulin, Michael Furian

**Affiliations:** 1 Department of Respiratory Medicine, University Hospital of Zurich, Raemistrasse 100, 8091 Zurich, Switzerland; 2 Department of Physiology and Pharmacology, Cumming School of Medicine, University of Calgary, Calgary, Canada; 3 Hotchkiss Brain Institute, Cumming School of Medicine, University of Calgary, Calgary, Canada; 4 Safety Group, Atacama Large Millimeter Submillimeter Array, Calama, Chile; 5 Department of Family Medicine, Cumming School of Medicine, University of Calgary, Calgary, Canada

**Keywords:** Medical research, Cerebrovascular disorders, Hypoxia, Environmental impact, Neuro-vascular interactions

## Abstract

Cerebral autoregulation (CA) is impaired during acute high-altitude (HA) exposure, however, effects of temporarily living high and working higher on CA require further investigation. In 18 healthy lowlanders (11 women), we hypothesized that the cerebral autoregulation index (ARI) assessed by the percentage change in middle cerebral artery peak blood velocity (Δ%MCAv)/percentage change in mean arterial blood pressure (Δ%MAP) induced by a sit-to-stand maneuver, is (i) reduced on Day1 at 5050 m compared to 520 m, (ii) is improved after 6 days at 5050 m, and (iii) is less impaired during re-exposure to 5050 m after 7 days at 520 m compared to Cycle1. Participants spent 4-8 h/day at 5050 m and slept at 2900 m similar to real-life working shifts. High/low ARI indicate impaired/intact CA, respectively. With the sit-to-stand at 520 m, mean (95% CI) in ΔMAP and ΔMCAv were − 26% (− 41 to − 10) and − 13% (− 19 to − 7), *P* < 0.001 both comparisons; mean ± SD in ARI was 0.58 ± 2.44Δ%/Δ%, respectively. On Day1 at 5050 m, ARI worsened compared to 520 m (3.29 ± 2.42Δ%/Δ%), *P* = 0.006 but improved with acclimatization (1.44 ± 2.43Δ%/Δ%, *P* = 0.039). ARI was less affected during re-exposure to 5050 m (1.22 ± 2.52Δ%/Δ%, *P* = 0.027 altitude-induced change between sojourns). This study showed that CA (i) is impaired during acute HA exposure, (ii) improves with living high, working higher and (iii) is ameliorated during re-exposure to HA.

## Introduction

Recent developments had led to an increase in the number of settlements and work places at altitude, especially, astronomical observation centers and resource extraction facilities that are often situated at very high altitudes (4000–5000 m). At the Atacama Large Millimeter/Submillimeter Array (ALMA), the largest ground-based telescope station worldwide, employees sleep at 2900 m and work at 5050 m for 7 days, and recover for 7 days at their permanent residence, normally near sea level. These repeated live high, work higher periods may have adverse effects on health, performance and safety of workers^1–3^.

Cerebral autoregulation (CA) protects the brain by a negative feedback loop mechanism, maintaining constant cerebral blood flow and oxygen delivery independent of fluctuations in systemic blood pressure^4^. Impaired CA results in systemic blood pressure associated cerebral blood flow fluctuations and may cause under-perfusion and inadequate oxygen delivery, or over-perfusion of capillaries with consequent disruption of the blood–brain barrier, capillary damage and micro-hemorrhages^5^. Furthermore, impaired CA has been shown to have consequences on the cerebral function and is related to cognitive impairment^5^.

At altitude, the arterial partial pressures of O_2_ and CO_2_, two key factors influencing CA functionality, are reduced^6^. Studies investigating CA functionality at different altitudes have reported persistent CA impairment with acute^7^ and prolonged high altitude exposure of up to 2 weeks compared to values reported near sea level^8^. However, real-life working shifts as implied in ALMA require workers to sleep at 2900 m and work at 5050 m. This pattern of exposure to high altitude (living high, working higher) induces an intermittent hypobaric hypoxic exposure, which might have beneficial effects on the CA functionality as suggested for intermittent hypoxic training in patients with Alzheimer diseases^9^. Furthermore, studies investigating the effects of repeated high altitude exposure showed milder reductions in O_2_ and CO_2_ when compared to previous altitude sojourns^2,10^. However, whether these improvements in arterial blood gases might have a beneficial effect on the CA has not been studied. Therefore, the purpose of this study was to test the hypotheses that CA is impaired during acute high-altitude exposure and that acclimatization (with an intermittent schedule of daily ascents to very high altitude, but sleeping at moderate altitude), and a 2nd altitude sojourn, ameliorates CA functionality compared to the first acute high altitude exposure. Furthermore, this study assessed whether altitude exposure has sustained effects on CA functionality after descending to low altitude.

## Methods

### Design and study setting

Data of the current study were collected within a prospective cohort study with the purpose to investigate the effects of acute, prolonged and repeated altitude exposure on cognitive performance in healthy subjects^2^. Results of the present study have not been published elsewhere.

**Figure 1:**
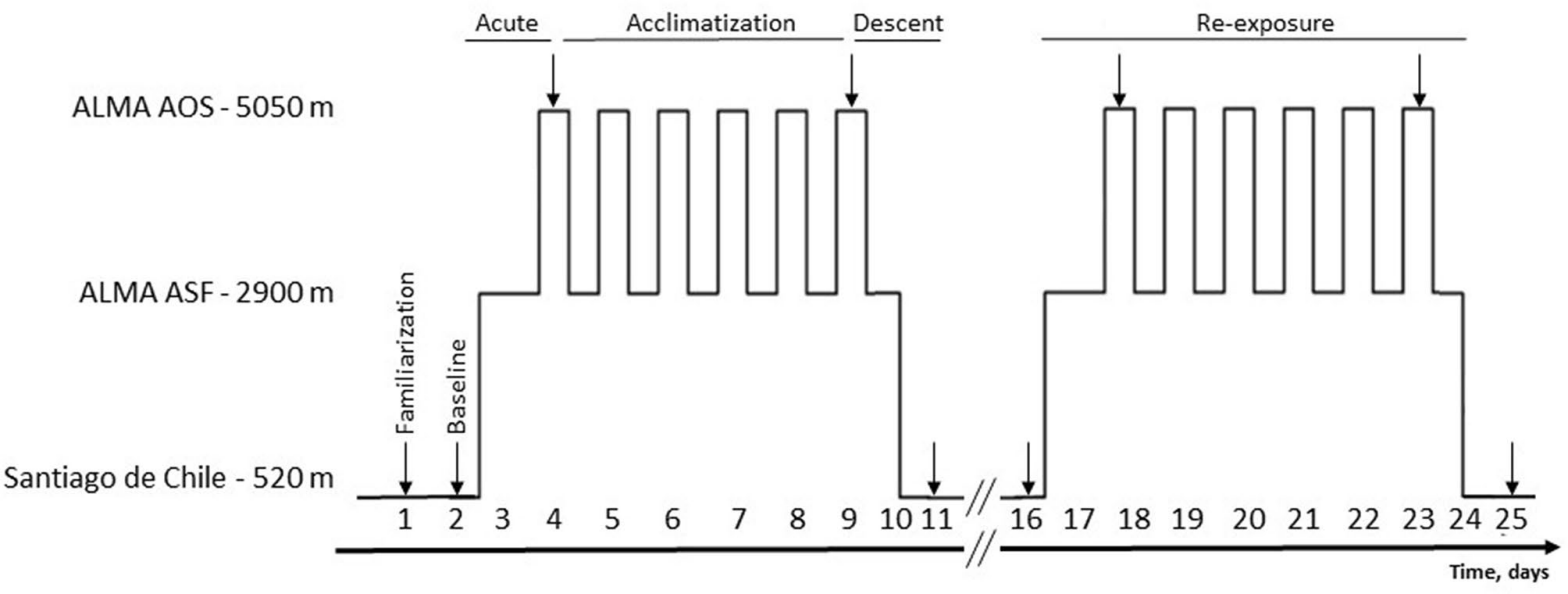
Study design. After a familiarization session at Santiago de Chile (520 m), participants underwent baseline assessments the day preceding travelling to high altitude. Participants travelled to the ALMA Operations Support Facility (ASF—2900 m) and stayed for one night before beginning daily sojourns to the Array Operations Site (AOS—5050 m). Participants alternated between 2900 m (sleeping) and 5050 m (up to 8 h/day). After a one-week recovery period at 520 m, participants performed a second, identical altitude cycle. Arrows indicate experimental testing days and headings indicate the condition of interest.

The ascent and assessment schedule is illustrated in Fig. 1. At Santiago de Chile (520 m), participants performed a familiarization and baseline session separated by a one-day rest interval. The day after, participants traveled by plane and bus (2 h each) to the ALMA Operation Support Facility (ASF; 2900 m) and spent seven consecutive nights at 2900 m. Each morning, participants were driven by car (45 min travel time) to the ALMA Operation Site (AOS; 5050 m), where they stayed up to 8 h without oxygen supplementation or any mediation against symptoms of acute mountain sickness. Measurements at 5050 m were performed on the 1st and 6th day and repeated on the day after descending to 520 m. This assessment schedule was repeated after a 7-day recovery period at 520 m. Measurements within each participant were conducted during the same time of day. To ensure balanced between-subject total exposure time to 5050 m, individual transportation schedules to 5050 m were applied. Participants were asked to refrain from alcohol throughout the expedition and to keep caffeine intake and exercise sequence and intensity as usual.

### Participants

Healthy, altitude-naïve men and women, aged between 18 to 30 years were recruited from the University of Calgary, Canada (N = 18, altitude of 1100 m). Participants were instructed to avoid any overnight stays at altitudes > 1500 m within four weeks before the study. All participants provided written informed consent. The study was approved by the Conjoint Health Research Ethics Board of the University of Calgary (Ethics ID: REB 15-2709) and the Cantonal Ethics Committee of Zurich (2016-00048). The main trial was registered at ClinicalTrials.gov (NCT02731456). All methods were carried out in accordance with relevant guidelines and regulations.

### Protocol and measurements

Sitting values were collected and averaged over 120 stable and artifact-free heart beats at the end of a 10-min sitting position in a comfortable chair. To assess CA functionality, participants stood up in less than 1 s and remained in standing position for 1 min^11^. The effect of standing up was measured by averaging the first 15 beats in standing position. This procedure was repeated and values were averaged within each subject. Due to the unknown effects of acute, prolonged and repeated exposures to high altitude on orthostatic tolerance and the blood pressure response to standing up, no threshold for a *successful* maneuver was applied (i.e. a minimum reduction of 15 mmHg from sitting to standing).

#### Clinical assessments

Clinical assessment included height, weight, oxygen saturation by pulse oximetry (SpO_2_) and heart rate measurements, as well as the environmental symptoms questionnaire cerebral score (AMSc) to assess acute mountain sickness (AMS)^12^. The AMSc comprises 11 questions on AMS symptoms (feel sick, feel hungover, coordination off, dim vision, lightheaded, headache, dizzy, loss of appetite, feel weak, nausea, faint) rated from 0 (not at all) to 5 (extreme). A weighted AMSc score ≥ 0.7 were assumed to indicate the presence of AMS.

#### Transcranial Doppler ultrasound

A 2-MHz Doppler probe (TOCM, Multigon Industries, New York, USA) was placed over the right temporal window to measure the peak blood velocity (MCAv) in the middle cerebral artery. The MCA was identified by systematic searches over the zygomatic arch and by varying the angle and position of the probe^13^. After adjustment and optimization of depth and angle of the probe, a marker was placed on the skin. A headband device was installed around the subject’s head and the Doppler probe was attached. This setting allowed optimal insonation position and angle during the complete protocol. To ensure a high standardization and reproducibility, the Doppler settings, probe position, insonation angle and depth were assessed during the familiarization period and the same settings were applied throughout the study. The cerebral autoregulation index (ARI) was defined by the ability of the CA to maintain a constant MCAv despite a rapid change in systemic blood pressure during a sit-to-stand maneuver. ARI was calculated as previously reported^14^:

$${\text{ARI}},\;\Delta \% {\text{MCAv }}/\Delta \% {\text{MAP }} = \frac{{\left( {{\text{MCAv }}\,{\text{standing}} - {\text{sitting}}} \right){ }/{\text{ MCAv }}\,{\text{sitting}}}}{{\left( {{\text{MAP }}\,{\text{standing}} - {\text{sitting}}} \right) / {\text{MAP }}\,{\text{sitting}}}}$$

$${\text{ARI}},\;\Delta \% {\text{MCAv }}/\Delta {\text{mmHg MAP }} = { }\frac{{\left( {\left( {{\text{MCAv }}\,{\text{standing}} - {\text{ sitting}}} \right){ }/{\text{ MCAv }}\,{\text{sitting}}} \right){ } \times { }100}}{{{\text{MAP }}\,{\text{standing }} - {\text{ sitting}}}}$$

Therefore, higher values in ARI represent larger MAP-induced MCAv fluctuations, corresponding to worse cerebral autoregulation, whereas smaller values in ARI corresponded to intact cerebral autoregulation. Cerebrovascular resistance and conductance indexes were calculated as MAP divided by the MCAv (CVRi) or vice versa (CVCi).

#### Additional physiological assessments

Continuous SpO_2_, heart rate and beat-by-beat systemic mean artery blood pressure (MAP) were assessed non-invasively by the finger-cuff technique (Finometer Midi, FMS, The Netherlands) and calibrated by brachialis sphygmomanometric measurements. Breath-by-breath end-expiratory partial pressure of CO_2_ (P_ET_CO_2_) and breathing frequency were assessed by capnography (Capnocheck Sleep, Smiths Medical PM Inc., Waukesha, Wi, USA). The capnograph was calibrated daily with a calibration gas containing 5% CO_2_ and the continuous P_ET_CO_2_ curve was re-calculated based on (if any) observed deviations.

### Outcomes

The main outcome of this study was the ARI (Δ%MCAv/Δ%MAP) and the effects of acute, prolonged and re-exposure to very high altitude. Additional outcomes were changes in explanatory cerebral, respiratory and cardiac variables. No a priori sample size estimation has been performed.

### Hypotheses

The three main hypotheses were that (i) acute high-altitude exposure impairs ARI (Δ%MCAv/Δ%MAP), (ii) 6 days of live high, work higher improves ARI (Δ%MCAv/Δ%MAP) and (iii) a 2nd compared to the 1st ascent to 5050 m has a lower impact on the altitude-related ARI impairment. An additional hypothesis was that CA functionality is immediately restored the day after descending to 520 m.

### Data analysis

The primary outcome was tested for normality by the Shapiro Wilk test. Due to the non-normally distributed data, mixed ordered logistic regression using the interaction between altitude (520 m and 5050 m), day at altitude (1st and 6th day) and number of altitude sojourns (1st and 2nd sojourn) as fixed effects, and participants as random effects was applied. Secondary outcomes were analyzed using mixed linear regression models. To avoid unnecessary multiple testing and false positive results, comparisons between day 6 at 5050 m vs. baseline (both, during the 1st and 2nd sojourn), descent vs. day 1 at 5050 m (both, during the 1st and 2nd sojourn) and baseline during the 2nd vs 1st sojourn were not statistically compared. To elucidate the underlying factors influencing ARI (Δ%/Δ%) or AMS, univariate and multivariate mixed linear regressions including baseline characteristics, SpO_2_, P_ET_CO_2_, MAP and MCAv were performed. Statistical significance was assumed when *P* < 0.05 and 95% confidence intervals of mean differences did not overlap zero.

## Results

A total of 18 healthy participants (11 women) with a mean age of 24 ± 4 years and BMI of 22.8 ± 3.1 kg/m^2^ were included in the final analysis. From a total of 216 planned 8 h sessions at 5050 m, 14 (6%) had to be terminated prematurely due to severe AMS symptoms. Participant characteristics are presented in Table 1. Cardiorespiratory and cerebrovascular outcomes are described in Table 2.

**Table 1:** Participant characteristics.

N	18
Men, (%)	7 (39)
Women, (%)	11 (61)
Age, years	24 ± 4
Body mass index, kg/m^2^	22.8 ± 3.1
Weight, kg	66 ± 9

Data are presented as mean ± standard deviation.

**Table 2:** Cardiorespiratory and cerebrovascular outcomes.

		Baseline, 520 m	1st sojourn		Descent, 520 m	Baseline, 520 m	2nd sojourn		Descent, 520 m
			1st day, 5050 m	6th day, 5050 m			1st day, 5050 m	6th day, 5050 m	
**Cardiorespiratory outcomes**									
Arterial oxygenation, %		96.5 ± 3.2	77.6 ± 3.2*	83.0 ± 3.2^§^	97.3 ± 3.2^^^	96.4 ± 3.2	81.3 ± 3.2*^$^	84.4 ± 3.2^§^	96.8 ± 3.2^^^
Heart rate, 1/min		70 ± 13	93 ± 13*	84 ± 13^§^	69 ± 13^^^	68 ± 13	89 ± 13*	84 ± 13^§^	71 ± 13^^^
Breath rate, 1/min		16 ± 4	16 ± 4	17 ± 4	17 ± 4	16 ± 4	16 ± 4	18 ± 4	17 ± 4
P_ET_CO_2_, mmHg		33.9 ± 2.5	24.5 ± 3.0*	24.4 ± 3.0	32.2 ± 2.5*^^^	33.9 ± 2.5	28.0 ± 0.7*^$^	26.9 ± 2.5	31.2 ± 2.5*^^^
Hemoglobin conc., g/dl		–	14.9 ± 1.3	15.4 ± 1.3^§^	–	–	15.2 ± 1.3^$^	15.8 ± 1.3^§^	–
AMSc score		0.12 ± 0.38	0.94 ± 0.38*	0.16 ± 0.38^§^	0.03 ± 0.38	0.15 ± 0.38	0.65 ± 0.42*	0.24 ± 0.38^§$^	0.02 ± 0.38^^^
**Cerebrovascular outcomes**									
Mean arterial pressure, mmHg	Sitting	87 ± 17	88 ± 17	89 ± 17	83 ± 17	81 ± 17	87 ± 17	91 ± 17	79 ± 17^^^
	Stand	73 ± 17	81 ± 17	79 ± 17	81 ± 17	75 ± 17	79 ± 17	85 ± 17	69 ± 17^^$^
	Diff	− 14 (− 23 to − 5)	− 7 (− 16 to 3)	− 10 (− 19 to 0)	− 3 (− 12 to 6)	− 5 (− 14 to 3)	− 7 (− 17 to 3)	− 6 (− 15 to 3)	− 10 (− 19 to − 1)
Mean MCA peak blood velocity cm/s	Sitting	57.9 ± 10.2	62.1 ± 10.2*	65.2 ± 10.2	58.0 ± 10.2^^^	56.2 ± 10.2	57.2 ± 10.2	60.3 ± 10.2	56.9 ± 10.2
	Stand	51.9 ± 10.2	54.3 ± 10.6	57.6 ± 10.2	51.9 ± 10.2^^^	50.1 ± 10.2	53.3 ± 10.6	53.7 ± 10.2	50.9 ± 10.6
	Diff	− 6.0 (− 9.9 to − 2.0)	− 7.8 (− 11.9 to − 3.7)	− 7.5 (− 11.5 to − 3.6)	− 6.1 (− 10.1 to − 2.2)	− 6.1 (− 9.9 to − 2.3)	− 4.0 (− 8.1 to 0.2)	− 6.6 (− 10.5 to − 2.6)	− 6.0 (− 10.4 to − 1.7)

		N = 15	N = 12	N = 14	N = 15	N = 16	N = 13	N = 15	N = 12
ARI, Δ%/Δ%		0.58 ± 2.44	3.29 ± 2.42*	1.44 ± 2.43^§^	1.40 ± 2.44 *	1.92 ± 2.36	1.22 ± 2.52^$^	1.63 ± 2.44	0.87 ± 2.42^$^
ARI, Δ%/mmHg		0.85 ± 2.90	3.50 ± 2.91*	1.35 ± 2.88^§^	1.72 ± 2.90	2.37 ± 2.80	1.43 ± 3.03^$^	2.24 ± 2.90	1.13 ± 2.91
Cerebrovascular resistance index, mmHg/cm/s	Sitting	1.55 ± 0.35	1.46 ± 0.31	1.42 ± 0.34	1.48 ± 0.35	1.48 ± 0.36	1.53 ± 0.36	1.57 ± 0.35	1.46 ± 0.31
	Stand	1.52 ± 0.35	1.53 ± 0.35	1.43 ± 0.34	1.68 ± 0.35^^^	1.55 ± 0.36	1.56 ± 0.36	1.67 ± 0.35	1.42 ± 0.35^^^
	Diff	− 0.03 (− 0.23 to 0.17)	0.07 (− 0.15 to 0.29)	0.01 (− 0.19 to 0.22)	0.20 (0.00 to 0.40)	0.08 (− 0.11 to 0.27)	0.03 (− 0.19 to 0.25)	0.11 (− 0.09 to 0.30)	− 0.04 (− 0.25 to 0.18)
Cerebrovascular conductance index, cm/s/mmHg	Sitting	0.67 ± 0.19	0.72 ± 0.21	0.74 ± 0.22	0.71 ± 0.23	0.71 ± 0.20	0.67 ± 0.22	0.67 ± 0.23	0.73 ± 0.21
	Stand	0.70 ± 0.23	0.71 ± 0.21	0.87 ± 0.22^§^	0.69 ± 0.23^^^	0.69 ± 0.20	0.67 ± 0.22	0.63 ± 0.23^$^	0.81 ± 0.21^^^
	Diff	0.03 (− 0.10 to 0.16)	0.00 (− 0.14 to 0.14)	0.13 (0.00 to 0.26)	− 0.03 (− 0.16 to 0.10)	− 0.20 (− 0.14 to 0.10)	0.00 (− 0.15 to 0.14)	− 0.04 (− 0.17 to 0.08)	0.08 (− 0.06 to 0.22)^$^
Systolic MCA peak blood velocity, cm/s	Sitting	91.4 ± 11.6	98.7 ± 10.4*	100.3 ± 11.2	95.0 ± 11.6	91.8 ± 12.0	93.5 ± 12.3	96.0 ± 13.2	93.9 ± 11.8
	Stand	95.6 ± 11.6	101.9 ± 10.4*	103.5 ± 11.2	98.0 ± 11.6	95.2 ± 12.0	97.5 ± 12.3	97.0 ± 13.2	95.8 ± 11.8
	Diff	4.2 (− 2.0 to 10.5)	3.2 (− 3.0 to 9.4)	3.2 (− 3.0 to 9.4)	3.0 (− 3.2 to 9.2)	3.4 (− 2.7 to 9.4)	4.0 (− 2.4 to 10.4)	1.0 (− 5.2 to 7.2)	1.9 (− 4.3 to 8.1)

Data are presented as mean ± SD or mean difference (95% CI). To avoid unnecessary multiple testing and false positive results, comparisons between day 6 at 5050 m vs. baseline (both, during the 1st and 2nd sojourn), descent vs. day 1 at 5050 m (both, during the 1st and 2nd sojourn) and baseline during the 2nd vs 1st sojourn were not statistically compared.

P_ET_CO_2_, end-tidal partial pressure of CO_2_; AMSc score, environmental symptom questionnaire cerebral score; MCA, middle cerebral artery; MAP, mean arterial pressure; ARI, autoregulation index.

*P < 0.05 versus 520 m baseline within sojourn; ^§^*P* < 0.05 between 6th versus 1st day at 5050 m; ^$^*P* < 0.05 when comparing 2nd versus 1st sojourn effects from acute (1st day 5050 m—520 m), prolonged (6th versus 1st day at 5050 m) or recovery (Descent 520 m—6st day at 5050 m); ^^^*P* < 0.05 between descent 520 m versus 6st day at 5050 m.

### Effect of acute altitude exposure (1st day at 5050 m compared to 520 m)

**Figure 2:**
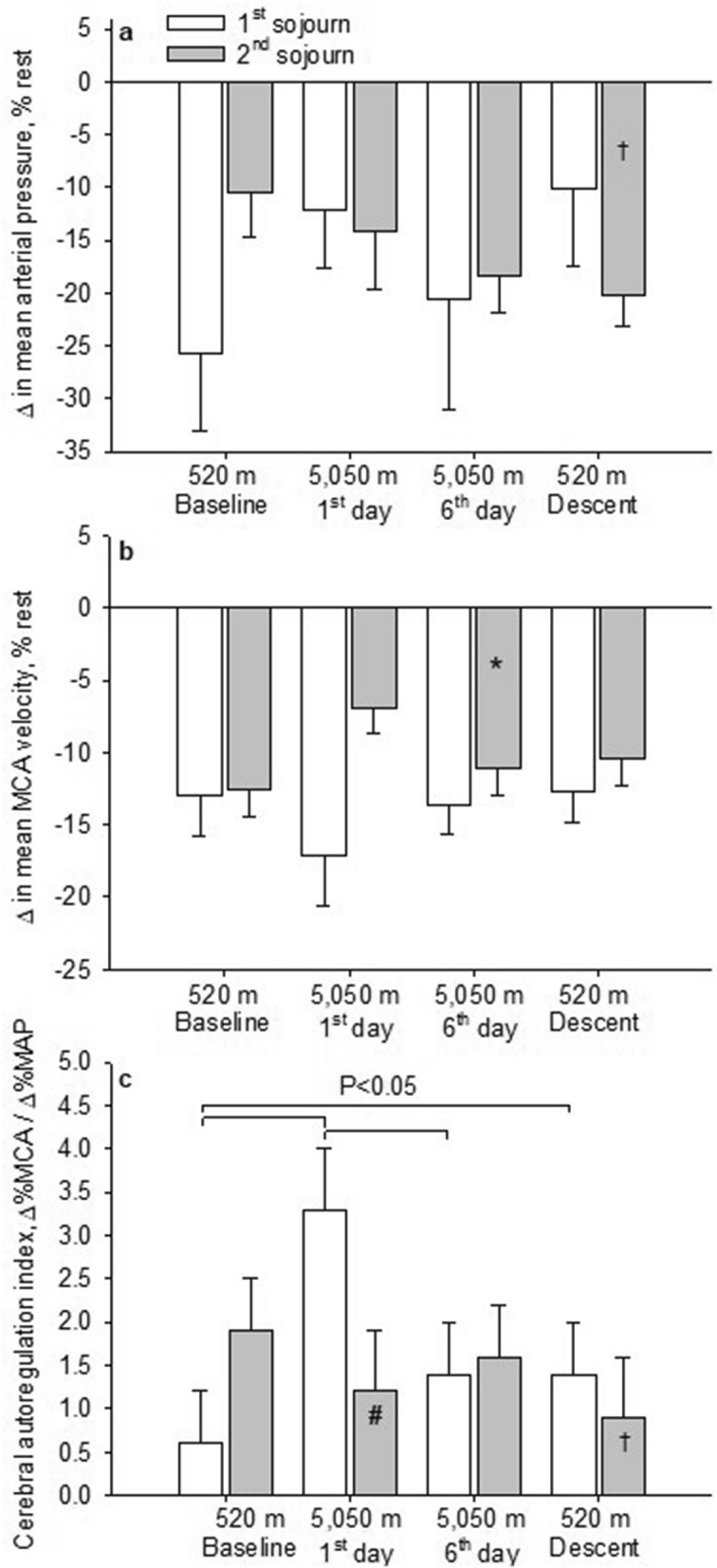
Cerebral autoregulation index assessed during a sit-to-stand maneuver. Panel (**A**) Mean arterial pressure (MAP) percentage change between standing versus sitting. Panel (**B**) Mean middle cerebral artery peak blood velocity (MCAv) percent change between standing versus sitting. Panel (**C**) Cerebral autoregulation index calculated by Δ%MCAv/Δ%MAP with a sit-to-stand maneuver. Standing values were obtained by averaging 15 beats following standing up < 1 s. ^#^*P* < 0.05 between the acute altitude-induced effect during the 2nd compared to the 1st sojourn; *P < 0.05 between the acclimatization effect during the 2nd compared to the 1st sojourn; †*P* < 0.05 between the descent effect during the 2nd compared to the 1st sojourn.

With acute exposure to 5050 m, participants had lower values in SpO_2_, mean ± SD, 77.6 ± 3.4% vs. 96.5 ± 3.4%, lower values in P_ET_CO_2_, 24.5 ± 3.0 mmHg vs. 33.9 ± 2.5 mmHg and higher heart rates, 93 ± 13 bpm vs. 70 ± 13 bpm and more symptoms of AMS assessed by the AMSc score, 0.94 ± 0.38 vs. 0.12 ± 0.38 compared to 520 m (*P* < 0.001 all comparisons). There was no acute altitude effect on MAP compared to 520 m (*P* = 0.939), whereas sitting mean MCAv significantly increased from 57.9 ± 10.2 cm/s to 62.1 ± 10.2 cm/s (*P* = 0.036) without altering CVRi or CVCi. The sit-to-stand maneuver at 5050 m caused a stronger drop in MCAv despite a milder drop in MAP, resulting in a significantly worse ARI compared to 520 m (3.29 ± 2.42 Δ%/Δ% vs. 0.58 ± 2.44 Δ%/Δ%, *P* = 0.007; 3.50 ± 2.91 Δ%/ΔmmHg vs. 0.85 ± 2.90 Δ%/ΔmmHg, *P* = 0.017) (Table 2, Fig. 2).

### Effect of prolonged intermittent altitude exposure (6th versus 1st day at 5050 m)

After 6 days of intermittingly ascending to 5050 m and sleeping at 2900 m, hemoglobin concentration and SpO_2_ were higher (83.0 ± 2.5% vs. 77.6 ± 2.5%, *P* < 0.001) and AMS symptoms lower, without altering P_ET_CO_2_ or breathing frequency compared to the 1st day at 5050 m. With acclimatization, MAP, MCAv, CVRi and CVCi remained unchanged. Compared to the 1st day at 5050 m, the sit-to-stand test caused a similar MCAv decrease despite a stronger drop in MAP, resulting in significantly improved ARI (Δ%/Δ%) compared to the 1st day at 5050 m (*P* = 0.039) (Table 2, Fig. 2).

### Effect of descent from high altitude (520 m versus 6th day at 5050 m)

On the day after descending from 5050 to 520 m, participants showed persistent P_ET_CO_2_ reductions (*P* = 0.027) and worse ARI (Δ%/Δ%) (*P* = 0.017) values compared to pre-ascent evaluations. After 1-week recovery period at 520 m, SpO_2_ and P_ET_CO_2_ recovered to pre-ascent values. ARI (Δ%/Δ%) tended to remain impaired but was not statistically different compared to pre-ascent assessments (*P* = 0.067).

### Effect of a second altitude sojourn (2nd compared to 1st sojourn at 5050 m)

Acute exposure during the 2nd compared to the 1st sojourn at 5050 m was accompanied with less hypoxemia and less hypocapnia. Heart rate similarly increased and breathing frequency remained unchanged as seen during the 1st sojourn at 5050 m. Compared to the 1st sojourn, MCAv did not increase during acute altitude exposure and ARI (Δ%/Δ%) deteriorated less compared to the 1st sojourn (Odds ratio of 0.10, 95% CI 0.01–0.76, *P* = 0.027) (Table 2, Fig. 2).

SpO_2_ and P_ET_CO_2_ remained higher after acclimatization in the 2nd compared to the 1st sojourn at 5050 m. This was accompanied with a further increase in hemoglobin concentration. MCAv, MAP and ARI (Δ%/Δ%) remained stable after acclimatization (Odds ratio for ARI of 7.3, 95% CI 0.95–56.7, *P* = 0.056) (Table 2).

When the participants descended from the 2nd sojourn at 5050 m, they persistently showed lower P_ET_CO_2_, lower MAP and better ARI (Δ%/Δ%) values compared to the values obtained on the day after descending from the 1st sojourn (Odds ratio of 0.12, 95% CI 0.02–0.82, *P* = 0.030).

### Predictors for AMS and ARI (Δ%/Δ%)

Predictors of AMS severity at 5050 m assessed by multivariate regression analysis were acute exposure, low sitting P_ET_CO_2_ and high ARI (Δ%/Δ%) values (Table 3). Only higher MAP values remained as an independent predictor of CA impairment defined by ARI (Δ%/Δ%) (Table 4).

**Table 3:** Predictors for acute mountain sickness severity at 5050 m assessed by mixed linear regression models.

Dependent variable: AMS-c score at 5050 m	Univariate				Multivariate			
	Coef	SE	95% CI	*P*	Coef	SE	95% CI	*P*
Acclimatization effect from 6th versus 1st day at 5050 m during the 1st sojourn	− 0.78	0.17	− 1.10 to − 0.45	< 0.001	− 1.00	0.19	− 1.38 to − 0.63	< 0.001
Re-exposure effect to 5050 m during the 2nd versus the 1st sojourn	− 0.28	0.17	− 0.61 to 0.05	0.096	− 0.21	0.21	− 0.61 to 0.20	0.313
Δ of the acclimatization effect during the 2nd vs. the 1st sojourn	0.36	0.24	− 0.10 to 0.82	0.128	0.58	0.26	0.07 to 1.09	0.026
Female sex	0.07	0.15	− 0.23 to 0.36	0.663				
P_ET_CO_2_, mmHg	− 0.05	0.02	− 0.10 to 0.00	0.036	− 0.09	0.03	− 0.14 to − 0.03	0.001
SpO_2_, %	− 0.02	0.02	− 0.05 to 0.01	0.192				
MCAv, ^cm^/_s_	0.00	0.01	− 0.02 to 0.01	0.891				
MAP, mmHg	0.00	0.01	− 0.02 to 0.01	0.684				
ARI, Δ%/Δ%	0.07	0.03	0.02 to 0.12	0.008	0.05	0.02	0.00 to 0.09	0.031
Intercept					3.19	0.72	1.79 to 4.59	< 0.001

N = 18 measured at 4 different time points (1st sojourn, 1st and 6th day at 5050 m; 2nd sojourn, 1st and 6th day at 5050 m). To account for the low number of observations (a total of 51 observations due to single missing values), the 5 most significant predictors from the univariate regression were entered into the multivariate regression model. Coefficients represent the change in the AMS-c score by changing one unit of the independent predictor.

AMS-c score = acute mountain sickness-cerebral score, P_ET_CO_2_ = partial pressure of end-expiratory carbon dioxide, SpO_2_ = oxygen saturation measured by finger pulse oximetry, MCAv = middle cerebral artery peak velocity, MAP = mean arterial pressure, ARI = autoregulation index.

**Table 4:** Predictors for cerebral autoregulation at 5050 m assessed by mixed ordered logistic regression models.

Dependent variable: ARI, Δ%/Δ%, quintiles	Univariate				Multivariate			
	Odds ratio	SE	95% CI	*P*	Odds ratio	SE	95% CI	*P*
Acclimatization effect from 6th versus 1st day at 5050 m during the 1st sojourn	0.29	0.21	0.07 to 1.19	0.087	0.29	0.24	0.06 to 1.48	0.138
Re-exposure effect to 5050 m during the 2nd versus the 1st sojourn	0.34	0.26	0.08 to 1.48	0.151	0.33	0.26	0.07 to 1.55	0.160
Δ of the acclimatization effect during the 2nd vs. the 1st sojourn	5.92	6.02	0.81 to 43.47	0.081	6.53	6.73	0.86 to 49.27	0.069
Female sex	0.87	0.43	0.33 to 2.28	0.773				
P_ET_CO_2_, mmHg	0.96	0.09	0.80 to 1.15	0.667				
SpO_2_, %	0.95	0.05	0.86 to 1.05	0.304	1.00	0.06	0.88 to 1.13	0.966
MCAv, ^cm^/_s_	1.03	0.03	0.98 to 1.08	0.307				
MAP, mmHg	0.96	0.02	0.91 to 1.00	0.060	0.95	0.02	0.90 to 1.00	0.039

N = 18 measured at 4 different time points (1st sojourn: 1st and 6th day at 5050 m; 2nd sojourn: 1st and 6th day at 5050 m). To account for the low number of observations (a total of 53 observations due to single missing values), the 5 most significant predictors from the univariate regression were entered into the multivariate regression model. Odds ratio represent the odds to change from one to the next higher ARI quintile by changing one unit of the independent predictor. Higher/Lower ARI quintiles represent worse/better cerebral autoregulation.

P_ET_CO_2_ = partial pressure of exhaled carbon dioxide, SpO_2_ = oxygen saturation measured by finger pulse oximetry, MCAv = middle cerebral artery peak velocity, MAP = mean arterial pressure, ARI = autoregulation index.

## Discussion

This study focused on the cerebral autoregulatory ability to protect the brain from rapid systemic blood pressure falls in maneuvers like the sit-to-stand test during acute, prolonged and repeated exposure to very high altitude (5050 m), a schedule often seen in high altitude shift workers, temporarily living high and working higher. The present findings suggest that cerebral autoregulation was impaired during acute exposure, but improved after a 7-day stay at high altitude (with a sleeping and working altitude of 2900 m and 5050 m, respectively). Strikingly, descending to lower altitude did not immediately improve CA and some impairments of the CA persisted. Moreover, novel findings of this study suggest that a 2nd altitude sojourn after one week at low altitude had a milder effect on the CA functionality, indicating that workers exposed to repeated high altitude exposures might be protected from initial CA impairments seen during the first acute high-altitude exposure. Exploratory analysis revealed that acute exposure to 5050 m, worse ARI (Δ%/Δ%) and lower P_ET_CO_2_ values were associated with higher AMS severity.

This study used a straightforward approach to describe CA proposed by a recent meta-analysis, by calculating the percent change in MCAv divided by the percent change from baseline in MAP^14^. The meta-analysis comprised 49 studies, whereas 41 studies used transcranial Doppler measurements to assess MCAv, a surrogate for cerebral blood flow. The average slopes proposed by the meta-analysis for a healthy population were 0.82 ± 0.77%ΔMCAv/%ΔMAP and 0.97 ± 0.91%ΔMCAv/ΔmmHg MAP. Therefore, the obtained baseline values at 520 m in our cohort were within the 95% confidence intervals of the cited study (Table 2, 0.58 ± 2.67%ΔMCAv/%ΔMAP and 0.85 ± 3.18%ΔMCAv/mmHg MAP). Furthermore, the current study used the sit-to-stand maneuver to provoke a change in MAP and MCAv. Although other interventions might have resulted in larger changes in these variables, the applied sit-to-stand maneuver showed a mean difference (95% CI) in MAP and MCAv at 520 m of − 26% (− 41 to − 10) and − 13% (− 19 to − 7) from sitting values, respectively. These changes are comparable with previous studies^11,15^. Notably, the effect of standing up on the CA was calculated by averaging 15 beats following the maneuver, and is in contrast to other studies choosing the nadir in MAP and MCAv following standing up^15^. However, by averaging 15 beats following the maneuver, findings remained less prone to artifacts and selection bias and were easily reproducible. In contrast, when averaging 15 beats, a shorter observation time of the CA due to higher heart rates at 5050 m was assessed and might have influenced our results.

Several previous field studies suggested that acute high-altitude exposure impairs CA functionality^3,16–19^. However, only a few studies have focused on the high-altitude acclimatization effect on the CA functionality. Subudhi et al. showed in 21 healthy subjects exposed to 3800–5260 m for 15 days that CA remained impaired compared to measurements near sea level and compared to the 1st day at 5260 m. This was confirmed by another study exposing 11 individuals for 4 weeks to 5260 m^17^. Both studies used transfer function analysis in comparison to the 2-point assessment of MCAv and MAP used in the current trial. However, these findings are in accordance with the current study showing that acclimatization does not restore sea-level values in CA.

The CA functionality depends on the ability to vasodilate or vasoconstrict cerebral arterioles in response to a stimulus. Acute high altitude exposure was associated with hypoxemia and hypocapnia, whereas hypoxemia presumably dominantly dilated cerebral arterioles, therefore, the MCAv increased during acute exposure to 5050 m. In the current study, 6 days of intermittently sleeping and working at 2900 and 5050 m, respectively, did improve arterial oxygenation by an increase in hemoglobin concentration and to a lower extend due to progressive hyperventilation, normally observed with altitude acclimatization. Whether the absent progression of hypocapnia is a result of the intermittent exposure schedule or due to lack of statistical power, remains to be elucidated. However, this finding is relevant since it has been shown that hypocapnia does augment CA functionality under hypoxic conditions^20^. Therefore, improvement of CA functionality with acclimatization seems to be more related to improved oxygenation than progressive hypocapnia. However, this study was not able to investigate other important factors potentially explaining alterations of CA functionality during acute or prolonged altitude exposure, i.e. altered CBF sensitivity to acute O_2_ and CO_2_ changes, sympathetic hyperactivity, alteration of endothelial function or neural nitric oxide synthase activity among others^7,17^.

In contrast, the current findings also support the thesis that acclimatization, at least partly, mitigated CA impairment, which was not observed in the two mentioned studies. A possible explanation might be the unique intermittent hypobaric hypoxic protocol used in this study (staying at 5050 m and sleeping at 2900 m), which might have altered autonomic nervous system activity, endothelial function or enhanced the sensitivity to hypoxia and hypocapnia, which might have improved the CA functionality over the time course of 6 days. Although, this has never been studied in detail, one trial applying 14 days of intermittent hypoxia (FiO_2_ 0.10, sessions of 5 × 4 min per day) showed diminished cerebral blood flow fluctuation to hypocapnia and hypercapnia after the intervention^22^, indicating improved CA functionality. Furthermore, beneficial effects of intermittent hypoxic training on the cerebrovascular response in Alzheimer patients have been reported^9^. This might explain the improved CA functionality with prolonged hypoxia during acclimatization. Other possible factors explaining the inconsistent findings of the acclimatization effect on CA between studies might be differences in the definition of “day 1” at high altitude, duration of the high altitude stay, intervention (sit-to-stand versus rest), analysis method (2-point assessment of MCAv and MAP versus transfer function analysis) or study protocol (staying at 5050 m and sleeping at 2900 m versus staying at 5260 m). Furthermore, in healthy lowlanders, it has been shown that the functionality of cerebral autoregulation is more protective to blood pressure elevations (CA ability to vasoconstrict blood vessels) than blood pressure reductions (CA ability to vasodilate)^14^. Since spontaneous blood pressure changes assessed by transfer function analysis includes both blood pressure elevations and reductions, this might provide different information about the CA functionality than exclusive blood pressure reductions induced by a sit-to-stand maneuver. This non-linear CA functionality might partly explain previously reported opposite findings at high altitude^8,23^. However, whether the altitude-induced CA impairment differs in the ability to protect the brain from blood pressure elevations versus blood pressure reductions has not been studied in detail.

Moreover, this study provides novel findings supporting the hypothesis that CA is less impaired during a second sojourn at very high altitude. Contrary to a previous study by Subudhi et al.^10^, which concluded that re-exposure to 5260 m after 7 days at sea level similarly impaired CA (based on transfer function analysis). Apart of the methodological differences outlined above, they have not performed a second baseline measurement and might have missed the circumstance that the participants had some retained CA impairments after 1 week at low altitude, as seen in the current study (Table 2). This would result in a different baseline and therefore, smaller altitude-induced difference in CA functionality. The observed smaller altitude-induced impact on CA functionality might be partly explained by preserved oxygen delivery towards the brain by elevated hemoglobin concentration, altered sensitivity to O_2_ and CO_2_, less altitude-induced hypoxemia, or by less vasodilatation of the small cerebral arteries allowing the CA to induce vasodilation in response to the sit-to-stand maneuver.

Whether the trend (*P* = 0.067) of persistent impairment of CA throughout 1-week post-altitude exposure was by chance or by lacking power to reach statistical significance requires further investigation. Findings on the day after the second descent to 520 m suggest already improved CA after the 2nd sojourn, indicating that CA functionality was normalized at low altitude after repeated exposure. Nevertheless, persistent impairment of CA after altitude sojourns would have an unknown impact on the safety and health of high-altitude workers and needs further investigation.

The assessment of the CA at high altitude remains challenging; many influential physiological parameters change, therefore, invasive and highly sophisticated measurements would be required and large sample sizes would be needed to account for the influence of various confounders. Often, changes of the MCAv assessed by TCD are assumed to reflect changes in cerebral blood flow. However, MCAv assessments at an altitude of 5050 m are influenced by changes of PaO_2_ and PaCO_2_, potentially changing the diameter of the MCA. In accordance, MCA diameter increases under isocapnic hypoxic condition (FiO_2_ 11%, equivalent to 5200 m)^24^ whereas the MCA cross-sectional area decreased after a reduction of P_ET_CO_2_ of 11–13 mmHg by voluntary hyperventilation (5.6–5.3 mm^2^, *P* < 0.01). However, 6/15 (40%) showed < 5% changes in the MCA cross-sectional area with hypocapnia^25^. In contrast, under hypocapnic hypoxic conditions seen at altitude, measurements have shown no^26^ or increased MCA diameter and cross-sectional area^21^. Therefore, one study in 24 participants applying 3-T Magnetic Resonance Imaging showed no changes of MCA diameter up to 5300 m (5.33 mm at sea level, 5.23 mm at 5300 m), and marked changes at 6400 m (6.66 mm), while another study found an increase in MCA diameter in 8 participants at 5050 m^21,26^. Although, the current study observed physiological changes in O_2_ and CO_2_ between altitudes, sojourns and with acclimatization, within the limits of the above mentioned studies (9.5 mmHg PetCO_2_ difference between baseline and day 6 at 5050 m during the first sojourn), no assessment of the MCA vessel caliber has been performed and any alterations in the MCA diameter cannot be ruled out. Nevertheless, the applied orthostatic challenge by the sit-to-stand maneuver is unlikely to have induced changes in the MCA diameter. Therefore, computed ARI values are reasonable assumed to reflect CA functionality. As mentioned before, this study applied a simple sit-to-stand maneuver to assess CA functionality rather than previously applied transfer function analysis, lower body negative pressure or injection of phenylephrine. The rationale was the practicality of the sit-to-stand test and the limited accessibility of the research facility at 5050 m as well as the rationale to provide data on the CA functionality representative for common daily activities of high altitude workers. In line with this rationale, calculation of the CA functionality by ARI (Δ%MCAv/Δ%MAP), suggested by Numan et al.^14^, was applied. Therefore, obtained high altitude ARI values can be compared to lowland reference values provided in the meta-analysis from Numan et al., avoiding differences in technical settings often seen during transfer function analyses (e.g. differences in sampling frequency, normalization, de-trending, filtering, window size). However, no threshold for a minimum MAP reduction with standing up was applied. The rationale was to avoid one-sided eliminations of potentially correct measurements due to improvements in orthostatic tolerance and blood pressure regulations. However, including maneuvers provoking only small MAP changes might have introduced some variability of ARI and might have resulted in lower statistical power to detect effects of acute, prolonged and repeated high altitude exposure on CA.

## Conclusions

First, this study confirmed CA impairment during acute exposure to very high altitude. Further, novel findings reported here are the partial improvements in CA over 7 days living high (sleeping at 2900 m) and working higher (5050 m). Additionally, we found a milder altitude-induced impact on the CA functionality during a 2nd similar sojourn at high altitude but a trend of persistent CA impairment up to 1-week post-altitude exposure, which resolved after the 2nd high altitude sojourn. Taken together, these findings suggest that workers commencing their high altitude work based on a *repeated living high and working higher *work schedule might be initially at risk for over and under-perfusion of brain areas, whereas staying and a 2nd working cycles at high altitude ameliorate CA functionality. Whether the improved CA functionality during the second high-altitude cycle indicates a protection also for several repeated work-shift cycles at high altitude remains to be determined.

## Data Availability

Anonymized data underlying this study can be requested by qualified researchers providing an approved proposal.

## Abbreviations

ALMA: Atacama Large Millimeter/Submillimeter Array
ALMA ASF: ALMA Operation Support Facility, 2900 m
ALMA AOS: ALMA Operation Site, 5050 m
AMS: Acute mountain sickness
AMSc: Environmental symptom questionnaire, cerebral score
ARI: Cerebral autoregulation index
CA: Cerebral autoregulation
CVCi: Cerebrovascular conductance index
CVRi: Cerebrovascular resistance index
MAP: Mean arterial blood pressure
MCA: Middle cerebral artery
MCAv: Middle cerebral artery peak blood velocity
P_ET_CO_2_: End-tidal partial pressure of carbon dioxide
SpO_2_: Arterial oxygen saturation measured by pulse oximetry
